# Portal lymphadenopathy predicts non-alcoholic steatohepatitis and advanced fibrosis in non-alcoholic fatty liver disease

**DOI:** 10.1371/journal.pone.0207479

**Published:** 2018-11-30

**Authors:** Saleh Daher, Namma Lev Cohen, Muhammad Massarwa, Mahmud Mahamid, Mira Nasser, Wadi Hazou, Rani Oren, Rifaat Safadi, Tawfik Khoury

**Affiliations:** 1 The Liver Unit, Institute of Gastroenterology and Liver Diseases, Department of Medicine, Hadassah University Hospital, Ein Kerem, Jerusalem, Israel; 2 The Department of Radiology, Hadassah University Hospital, Ein Kerem, Jerusalem, Israel; 3 Gastroenterology and endoscopy united, The Nazareth Hospital, EMMS, Nazareth, Israel; University of Tsukuba, JAPAN

## Abstract

**Background and aim:**

The progression of non-alcoholic fatty liver disease (NAFLD) to non-alcoholic steatohepatitis (NASH) is believed to be the driver for future development of fibrosis and cirrhosis. Nevertheless, there remains a clear deficit in non-invasive methods for the diagnosis of NASH. The aim of the present study was to evaluate the prevalence of portal lymphadenopathy (PL) in biopsy- proven NAFLD patients and to determine whether PL correlates with NAFLD stage and severity.

**Methods:**

A retrospective study included biopsy-proven NAFLD patients with up to date (within one year) abdominal imaging by computed tomography (CT) and/or magnetic resonance imaging (MRI). Patients were clustered into three groups based on their NAFLD Activity Score (NAS): NAS1-2 (mild), NAS3-4 (moderate) and NAS≥5 (advanced). We Assessed for association between PL and other clinical and laboratory findings with NAS, NAS components and fibrosis.

**Results:**

Seventy-five patients with NAFLD and no other competing etiologies for liver diseases or PL were included. The mean age was 50.7±14.84 years with male predominance (N = 47, 62.7%). Twenty-five (33.3%), 37 (49.3%) and 13 (17.3%) patients had mild, moderate and advanced NAS, respectively. PL significantly correlated with advanced NAS ≥ 5 (Fisher’s (F) 9.5, P = 0.009). Correlation was driven mainly by a link to hepatocytes ballooning (F of 5.9, P = 0.043). In addition, PL significantly correlated with portal inflammation (F 4.29, P = 0.038). As for hepatic fibrosis, the F test wasn’t significant, though spearman’s coefficient (SC) was significant (0.277, P = 0.012). On multivariate analysis, PL was identified as a sole predictor of advanced NAS score (Odds ratio of 2.68, P = 0.002). Incorporation of PL into noninvasive fibrosis scores improved their diagnostic yield.

**Conclusion:**

PL predicts severity of NAFLD. Its presence may serve as a novel radiological marker for NAFLD/NASH differentiation and disease progression.

## Introduction

Non-alcoholic fatty liver disease (NAFLD) is characterized by hepatic fat accumulation and is largely a manifestation of metabolic syndrome [1]. The reported prevalence of NAFLD in obese people ranges between 75–100% [2]. Several studies have shown that almost 30% of the general population worldwide have radiological signs of fatty liver [3] [4] [5]. Currently, NAFLD is considered the most common cause worldwide for the development of chronic liver disease and liver cirrhosis [6]. The spectrum of NAFLD ranges from simple steatosis or isolated fatty liver, which is hepatic fat infiltration without inducing inflammation and without hepatocellular injury, to non-alcoholic steatohepatitis (NASH), characterized by hepatic fat infiltration coupled with hepatocyte inflammation. Studies have shown that approximately 20–30% of patients with NAFLD develop NASH, which is associated with increased morbidity and mortality [7]. NASH, in contrast to simple steatosis, was shown to be associated with an increased risk for development of cirrhosis and hepatocellular carcinoma (HCC) [8] [9] [10] [11].

To date, there is a scarcity of non-invasive tools that can predict the progression of NAFLD. The only reliable predictor is liver biopsy, an invasive and costly procedure [12].

The hepatic lymph originating in the sinusoids flow through the space of Disse and follows two major lymphatic pathways–the first traveling along the portal tracts to the hilum of the liver, and the second traveling along central veins and draining into mediastinal lymph nodes [13], the latter being the dominant through which drainage of about 80% of the hepatic lymph occurs. Normal hepatic lymph structures cannot be visualized by imaging, unless there is an underlying disease in proximity such as hepatic lymphoproliferative disease, hepatic lymphangitis, carcinomatosis or lymphocele, which are associated with the development of portal hepatic lymphadenopathy [14].

The aim of this study was to examine the prevalence and clinical significance of portal lymphadenopathy (PL) in NAFLD patients, and to determine whether its presence correlates with NAFLD severity, as assessed by NAFLD activity score (NAS), as well as with liver fibrosis.

## Materials and methods

### Study population

Study population was comprised of adult male and female patients who were diagnosed with NAFLD by liver biopsy between the years 2006 and 2016 and who had a computed tomography (CT) or a magnetic resonance imaging (MRI) imaging performed within 12 months before or after the performance of liver biopsy. We excluded patients with other causes of liver diseases, such as autoimmune hepatitis, viral hepatitis or who reported consumption of > 30 grams of alcohol per day. Patients who were treated with vitamin E or participated in clinical trials, had malignancy within 5 years before liver biopsy, had other causes of lymphadenopathy, such as infectious, neoplastic or autoimmune diseases, and pregnant patients at time of imaging or biopsy were all excluded.

### Data collection

Imaging files and pathology slides were retrieved from the central radiology system and the pathology department archive of the Hadassah university hospital which is a tertiary referral center. Blood tests results were retrieved from patients records and were all performed at time of liver biopsy. Patients demographic and clinical data were retrieved from the electronic patient record.

### Study design

A retrospective, cross sectional design was utilized. All pathology slides and imaging files were reviewed by a blinded senior radiologist and pathologist. NAS was used to evaluate NASH severity according to three components: 1) Steatosis, 2) Hepatocytes ballooning and 3) Lobular inflammation [15]. Patients with portal lymph nodes larger than 10 mm were considered to have PL. Included patients were clustered into three groups according to their NAS. Patient with NAS of 1–2, likely to have simple steatosis without steatohepatitis, were labeled as "***mild disease"***, those with NAS 3–4, likely to have steatohepatitis, as "***moderate*** disease", and those with NAS of 5–8, likely with ***advanced*** steatohepatitis, as severe disease [16]

We assessed association between PL and other clinical and laboratory findings with NAS, with each of its components, with portal inflammation and with hepatic fibrosis. Dependent variables were patients laboratory, imaging (presence of lymphadenopathy) and clinical characteristics while independent variables were NAS, its components, portal inflammation and fibrosis stage. The Hadassah-Hebrew university medical center institutional review board (IRB) approval was obtained. Trial registration number: 0131-16-HMO. Written informed consent was waived by the IRB due to the retrospective non-interventional design of the study.

## Statistical analysis

Characteristics of participants were presented with descriptive statistics as arithmetic means (±SD) or range for continuous variables, or as percentages for categorical variables. Association between continuous variables (patients' blood tests and calculated scores) and histological findings was measured by Pearson correlation and were validated by Mann-Witney when the distribution of the continuous variables was not normal.

The association between categorical variables (background illness and presence of lymphadenopathy vs. histological findings) was tested with χ2 and Fisher’s exact test as appropriate. We used Generalized Linear Models with lymphadenopathy as the dependent binary logistic variable. The non-lymphadenopathy participants served as the control group. A multivariate analysis included age and suspected confounders that showed an association with NAS or one of its components in the univariate analysis at the p < .05 level of significance. Linear regression was utilized to formulate a formula implementing lymphadenopathy into the traditional fibrosis scores. Analyses were done with IBM-SPSS software, version 23.

## Results

### Baseline characteristic of the patient’s cohort

Seventy-five patients with biopsy-proven NAFLD were included in the study. Twenty-five patients (33.3%) had mild disease (NAS 1–2), thirty-seven (49.3%) had moderate disease (NAS 3–4) and thirteen (17.3%) had advanced disease (NAS ≥ 5). The mean ± standard deviation (SD) for age of study subjects was 50.73±14.84 (range 18–81). Forty-seven patients (62.7%) were male. The average age and the male\female ratio were similar among all the 3 NAS groups (P = NS). PL as per imaging was found in 32/75 (42.7%) of patients (Table 1). Comparing patients with PL with those without PL, we found that the averages of NAS score, HB, LI and PI were significantly higher among patients with PL. Table 2 show baseline characteristic of positive PL population as compared to negative PL.

**Table 1:** Clinical and demographic characteristics of study participants.

**Patients number**	75
**Mean age ± SD (range)**	50.73±14.84 (18–81)
**Male\Female**	47\28
**HTN N(%)**	20(26.7)
**T2-DM N(%)**	22(29.3)
**Hyperlipidemia N(%)**	16(21.3)
**Cirrhosis N(%)**	15(20)
**IHD N(%)**	8 (10.7)
**PL N(%)**	32 (42.7)

**Table 2:** Baseline characteristics of PL+ and PL- groups.

	PL +	PL -	P value
**Number**	32	43	
**Mean age ± SD (range)**	50±15.3 (19–78)	51.2±14.6 (15–81)	0.3
**Male\Female N**	20\12	27\16	0.48
**HTN N(%)**	13 (40.6)	5 (11.6)	0.033
**T2-DM N(%)**	12 (37)	12 (27.9)	0.1
**Hyperlipidemia N(%)**	7 (21.8)	7 (16.3)	0.2
**Cirrhosis N(%)**	8 (25)	8 (18.6)	0.4
**IHD N(%)**	7 (21.8)	2 (4.6)	0.06
**Mean ALT (U/L) ± SD (range)**	51.3±45.4 (8–236)	52.6 ±46.3 (8–226)	0.45
**Mean AST (U/L) ± SD (range)**	45.7±29.2 (15–137)	50.9±50 (9–265)	0.33
**Mean GGT (U/L) ± SD (range)**	112.6±134.2 (16–618)	96.5±78 (20–348)	0.28
**Mean ALK (U/L) ± SD (range)**	91.1±38.5 (45–201)	108.3±96.5 (11–659)	0.17
**Mean T. Bili (umol/L) ± SD (range)**	20.3±25.7 (3–137)	15.7±16.3 (3–84)	0.17
**Average NAS score (range)**	3.62 (1–6)	2.74 (1–6)	0.003
**Average steatosis score (range)**	1.84 (1–3)	1.6 (0–3)	0.08
**Average HB (range)**	1.06 (0–2)	0.67 (0–2)	0.008
**Average LI (range)**	0.72 (0–2)	0.46 (0–1)	0.02
**Average fibrosis (range)**	2.25 (0–4)	1.53 (0–4)	0.008
**Average PI (range)**	1.12 (0–2)	0.81 (0–2)	0.008

### NAS distribution and laboratory findings

The distribution of NAS, NAS components, fibrosis stage and laboratory findings are shown in Table 3. The NAS in our cohort ranged from 1–6 as no patient had a NAS of 7 and 8 points. The mean NAS was 3.12±1.4. The mean ± SD for steatosis, hepatocytes ballooning (HB), lobular inflammation (LI), fibrosis and portal inflammation (PI) grade in our cohort were 1.71 ± 0.73, 0.84 ± 0.7, 0.57 ± 0.57, 1.84 ±1.28 and 0.95 ± 0.56, respectively (Table 3).

**Table 3:** Descriptive statistics of NAS, laboratory findings and fibrosis scores.

Parameters	Mean (±SD)	Range
**NAS**	3.12 ± 1.4138	1–6
**Steatosis**	1.71 ± 0.73	0–3
**HB**	0.84 ± 0.7	0–2
**LI**	0.57 ± 0.57	0–2
**Fibrosis**	1.84 ± 1.28	0–4
**PI**	0.95 ± 0.56	0–2
**ALT(U/L)**	52.08 ± 45.6	8–236
**AST (U/L)**	48.54 ± 42.05	9–265
**ALK (U/L)**	100.79 ± 76.75	11–659
**GGT (U/L)**	104.14 ± 107.65	16–618
**T. Bili (umol/L)**	17.74 ± 20.93	3–137
**WBC (10**^**9**^**/L)**	7.09 ± 2.12	3.2–16.4
**HgB (gr %)**	13.51 ± 1.9	7.8–17.2
**INR**	1.19 ± 0.3	1–2.4
**Plts (10**^**9**^**/L))**	189.45 ± 84.27	33–457
**CRP (mg %)**	0.73 ± 0.76	0–3
**FIB-4 score**	3.11 ± 2.91	0.45–12
**APRI score**	0.92 ± 0.71	0.1–3.1

### Association of demographic, clinical and laboratory findings with NAS and its sub-scores

The results of univariate analysis are shown in Table 4. Age was found to be negatively correlated and alanine aminotransferase (ALT) positively correlated with steatosis (P = 0.022 and P = 0.004, respectively). Higher total bilirubin (TB), international normalized ration (INR) and C-reactive protein (CRP) and reduced platelet (PLT) count were all associated with increased HB (P = 0.025, P = 0.027, P = 0.026 and P = 0.013, respectively). Higher INR, white blood cells (WBC) and lower hemoglobin (HgB) were positively correlated with LI (P = 0.027, P = 0.048 and P = 0.001, respectively). Furthermore, higher Age, TB, and INR levels correlated positively with advanced stages of fibrosis (P = 0.013, P = 0.001 and P = 0.001 respectively), while ALT, Hgb and PLTs negatively correlated with higher stages of fibrosis (P = 0.017, P = 0.013 and P = 0.0001, respectively). There was a trend for correlation between age and ALT with higher NAS (P = 0.056 and P = 0.07, respectively) (Table 4). We found no significant correlation between background illness–ischemic heart disease (IHD), Type 2 Diabetes Mellitus (T2-DM), hypertension (HTN) and dyslipidemia and NAS. On the other hand, T2-DM had significant correlation with fibrosis stage (P = 0.027) and borderline correlation with LI (P = 0.051). HTN was found to correlate with LI (P = 0.04).

**Table 4:** Correlation of laboratory variables and age with NAS, NAS components, fibrosis stage and portal inflammation- univariate analysis.

		AGE	ALT	AST	GGT	ALK	T. Bili	INR	CRP	WBC	HgB	PLT
**NAS**	Pearson	-.221	.214	.199	-.057	-.082	.195	.174	.263	.209	-.220	-.096
	p*	0.056	0.070	0.165	0.676	0.491	0.098	0.139	0.122	0.075	0.060	0.413
**HEPATOCYTE BALLOONING**	Pearson	-.102	.029	.056	-.170	-.138	.262	.257	.372	.077	-.134	-.287
	p*	0.384	0.808	0.698	0.207	0.244	.025	.027	.026	0.513	0.255	0.013
**LOBULAR INFLAMMATION**	Pearson	-.085	.060	.180	.160	.093	.207	.258	.069	.231	-.366	-.017
	p*	0.468	0.617	0.211	0.236	0.436	0.078	.027	0.690	0.048	0.001	0.887
**STEATOSIS**	Pearson	-.264	.336	.200	-.087	-.099	-.036	-.113	.109	.145	-.006	.101
	p*	0.022	0.004	0.164	0.520	0.402	0.765	0.337	0.526	0.218	0.957	0.391
**FIBROSIS STAGE**	Pearson	.286	-.279	-.058	.069	-.070	.367	.675	.271	-.055	-.288	-.532
	p*	0.013	0.017	0.689	0.607	0.559	0.001	0.001	0.110	0.640	0.013	0.001
**PORTAL INFLAMMATION**	Pearson	.165	-.108	-.139	.027	-.146	.071	.254	.111	-.296	-.061	-.323
	p*	0.156	0.361	0.334	0.840	0.217	0.551	0.029	0.521	0.01	0.606	0.005

*2 tailed P value

green shadowing indicates positive correlation, red shadowing indicates negative correlation

### Association of portal lymphadenopathy with NAS, portal inflammation and fibrosis

**Fig 1:**
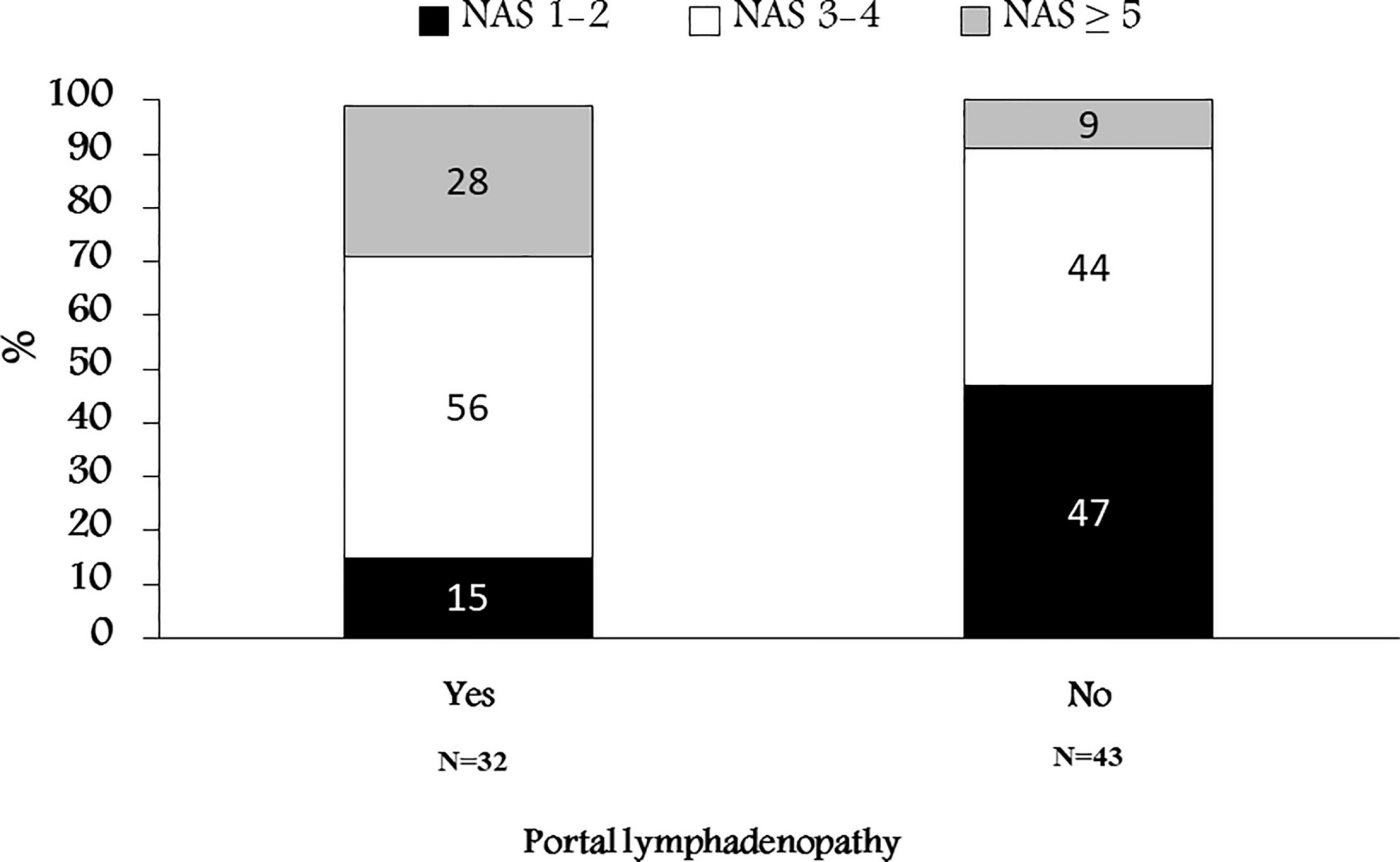
Distribution (%) of NAS according to the status of Portal lymphadenopathy.

On univariate analysis, there was a significant association between the presence of PL and the severity of NAS (Fig 1, Table 5) (P = 0.009). Only 9% of patients without PL had advanced NAS compared to 28% of patients with PL. Of patients with positive PL, only 15% had mild NAS. The discriminative power of PL was low in the moderate NAS, as 44% did not have PL as compared to 56% who had PL. When further analyzing the correlation with the NAS sub-scores, we found a statistically significant correlation between PL and HB (P = 0.043), as only 18.8% of patients with PL didn’t had HB as compared to 44.2% of patients without PL who didn’t have HB. Notably, there was only a trend for correlation between PL and LI (P = 0.075), while no correlation was found with steatosis (P = 0.1). The correlation between PL and fibrosis was not significant as per Fisher’s test (P = 0.146) but spearman’s coefficient showed significant correlation (P = 0.012), implying some relation do exist (Table 5). By performing further analysis utilizing one-way-Anova, a positive correlation between PL and fibrosis was clearly seen (P = 0.016).

**Table 5:** Correlation of PL with NAS, NAS components and fibrosis-univariate analysis.

		NAS	HEPATOCYTE BALLOONINBG	LOBULAR INFLAMMATION	STEATOSIS	HEPATIC FIBROSIS
**PORTAL LYMPHADENOPATHY**	FISHER'S	9.566	5.980	4.700	3.500	6.671
	p	**0.009**	**0.043**	0.075	0.296s	0.146
	SPEARMAN’S COEFFICIENT	0.356	0.280	0.198	0.174	0.292
	p	**0.002**	**0.015**	0.086	0.143	**0.012**

### Multivariate analysis

All parameters that were significantly correlated with NAS sub-scores or showed a trend for association with NAS as assessed by univariate analysis were included in the multivariate model (Table 6). PL was identified as the only significant predictor of advanced NAS (Odds ratio (OR) = 2.68, P = 0.002) (Table 6). Not being diagnosed with T2-DM had a protective effect against high NAS (OR = 0.027, P = 0.01). Increased age and PLT count were borderline associated with decreased NAS. PL was also significantly associated with the presence of PI (OR = 1.29, P = 0.033) and hepatic fibrosis (OR = 1.5, P = 0.033) but showed only a trend for association with HB (P = 0.08) and LI (P = 0.06). PL was not associated with degree of steatosis (P = 0.11).

**Table 6:** Correlation of demographic, clinical and laboratory variables with NAS -Multivariate analysis.

	Sig.*	EXP(B)^$^	95% Confidence Interval
**AGE**	**0.01**	0.97	0.94–0.99
**WBC**	0.23	1.09	0.95–1.26
**HgB**	0.91	0.99	0.80–1.22
**Plts**	**0.01**	0.99	0.99–1.00
**INR**	0.85	0.89	0.26–3.06
**PL**	**0.002**	2.68	1.42–5.07
**ALT**	0.93	1.00	0.99–1.01
**TB**	0.24	1.01	0.99–1.03
**CRP**	0.43	1.22	0.74–2.03
**[T2-DM = 0]**^**¥**^	**0.01**	0.27	0.10–0.71
**[T2-DM = 1]**		1.00	
**[HTN = 0]**^**¥**^	0.20	1.89	0.72–4.95
**[HTN = 1]**		1.00	

* Wald Chi-Square

¥ 0 denotes patient does not have disease

$ signifies odds ratio

### Correlation of portal lymphadenopathy with laboratory findings and demographics

We found a significant correlation between PL and HTN (OR = 3.5, P = 0.033). While, only a trend for correlation was seen with IHD (OR = 4.7, P = 0.06). However, no correlation was seen with age, T2-DM and hyperlipidemia (P = NS). There was no correlation between HTN and fibrosis (P = 0.6). In the other hand, we found that HTN correlated with LI (P = 0.04), while no correlation was seen with steatosis or HB (P = 0.4). Additionally, the presence of PL didn’t correlate with liver enzymes, TB and white blood count, while we noticed a decreased PLT count in patients with PL (P = 0.05). No correlation between CRP and PL was noted.

### Diagnostic performance for NASH

Using a NAS cutoff of ≥3, the sensitivity, specificity, positive predictive value (PPV) and negative predictive values (NPV) for the diagnosis of NASH stood at 54%, 80%, 84% and 46%, respectively. Using a cutoff of > = 5 yielded sensitivity, specificity, PPV and NPV of 69%, 62%, 28% and 91%.

### Impact on the diagnostic yield of fibrosis scores

The ALT to PLT ratio (APRI) score and Fibrosis-4 (FIB4) score were found to significantly correlate with fibrosis stage (Pearson’s coefficient 0.42 and 0.52, respectively, both P<0.001). Via a generalized linear model, we found that addition of the status of PL (absent = 0 or present = 1) to APRI score significantly enhanced the diagnostic yield. Having a high APRI score, presence of PL increased the odds for hepatic fibrosis (Regression coefficient of 0.575, P = 0.45). The status of PL can be incorporated in a new regression equation: 0.963+0.688*APRI+0.575*PL (1 or 0). This equation require validation in further prospective trials. As for FIB-4 the increment in diagnostic yield didn’t reach statistical significance.

## Discussion

In the present study, we found that the presence of PL as assessed by CT/MRI imaging, strongly correlated with a more advanced stage of NAFLD, as defined by NAS of ≥ 5. Furthermore, within the NAS components, we found that PL significantly correlated with HB, while only a trend was seen with LI and no correlation was seen with simple steatosis. Using a NAS cutoff of 5, the status of PL can be utilized to rule out NASH with a NPV of 91%. Addition of the status of PL improves the diagnostic yield of non-invasive fibrosis scores.

The spectrum of NALFD ranges from simple hepatic steatosis, which usually remains stable over the years, [17] [18], to steatohepatitis and subsequent liver cirrhosis and HCC, with increased mortality if liver transplantation is not performed [19] [20] [21]. While patients with simple steatosis do not require close observation, patients with steatohepatitis and fibrosis require close monitoring for the development of liver cirrhosis. Steatohepatitis signifies a transition state from relatively benign isolated fatty liver to parenchymal inflammation and scar formation. Thus, identifying the presence and severity of liver inflammation in patients with NAFLD is of critical importance in guiding the subsequent disease management. Currently, the only mean of accurately assessing the degree of hepatic inflammation and fibrosis in NAFLD patients is via liver biopsy. Several non-invasive, non-radiological scoring scales for NAFLD, including NAFLD fibrosis score [15], APRI score [22], FIB-4 index [23] and BARD score [24] have recently been incorporated into clinical practice, but still suffer from some limitations and are used to assess the probability of fibrosis, not inflammation [25].

A recent study claimed that the presence of HB provides a more confident diagnosis of NASH [26]. Interestingly, while PL clearly associated with NAS, on univariate analysis the only significant association with NAS components was with HB with only a trend towards association with LI. However on multivariate analysis the association was a bit weaker and below significance. A previous study showed a positive correlation between CRP level and histologically proven hepatic inflammation [27]. In the present study, CRP correlated only with HB, but not with NAS.

The observed correlation between PL and PI coupled with the similar CRP level among the three NAS groups and the absence of leukocytosis might suggest that the presence of PL is secondary to the development of steatohepatitis and not secondary to systemic inflammatory reaction. We also showed that PL significantly correlated with advanced fibrosis stage as being assessed by histology and by the non-invasive FIB-4 and APRI scores. These score are limited by their sensitivity and specificity [23]. In our study we showed that the addition of PL to APRI score significantly enhance the diagnostic performance of fibrosis. Thus, it might be worthful to incorporate PL in those scores.

An unexplained finding was the correlation between systemic arterial hypertension and PL. Recent studies reported that the incidence of HTN increases in NAFLD severity stage and its rate is gradually increased with advancing NAFLD [28]. In our study, we assume that the correlation between systemic arterial HTN and PL is an indirect sign of advanced NASH stages since PL was shown to be a strong predictor of more severe form of fatty liver disease, thus, HTN is a comorbid disease associated with advanced NASH rather than a cause of PL. In addition, we found that the grade LI was higher in both T2-DM and HTN, while the stage of fibrosis was associated with the diagnosis of T2-DM, thus emphasizing the need for in-depth evaluation of NAFLD in patients with T2-DM and HTN. PL didn’t correlate with liver enzymes (ALT, aspartate transaminase (AST), gamma-glutamyl transferase (GGT), alkaline phosphatase (ALK), TB) and WBC. Notably, there was a strong trend for correlation with low PLT count as compared to patients without PL (P = 0.05). This observation might also underscore the importance of PL as a predictor for advanced NAFLD and fibrosis stage which might reflect the development of subclinical portal hypertension as manifested by decreased PLT count.

The non-invasive evaluation scales are typically used to evaluate fibrosis in NASH patients, and there remains a need for scores evaluating the inflammatory component in NAFLD/NASH. Previous animals studies have shown that lipopolysaccharides were elevated in animal models of NAFLD with progression to NASH [29] [30]. Previously, ALT has been used as an indicator of NASH [31]. In our study we showed that ALT but not AST correlated with higher NAS score ≥5 on univariate, but not multivariate analysis. However, previous study has questioned the predictive role of ALT for steatohepatitis and showed that even patients with NASH had normal ALT level [32]. Thus, the role of liver enzymes in histological prediction of NAFLD is still debated. Other biochemical markers (higher TB level and INR) were associated with advanced fibrosis and NAS score.

To date, there have been no reports addressing PL in predicting evolution from NAFLD to NASH. In our study, we found that PL predicts more severe hepatocytes ballooning, NASH as assessed by advanced NAS score ≥ 5, higher PI and fibrosis grades.

The present study had several limitations. It was a retrospective study with a relatively small sample size. A major limitation for every day practice is that ultrasonographic assessment of portal lymphadenopathy was not evaluated. Several laboratory parameters were missing for some patients. Finally, the study was performed in a single center. In the other hand, the definite diagnosis of NASH by means of liver biopsy assessed by senior liver pathologist, stood as the main strength of our study.

In conclusion, portal lymphadenopathy, as assessed by CT/MRI, is significantly associated with advanced stages of NAFLD, as reflected by NAS score and by fibrosis stage, and with PI, HTN and low PLT count–all pointing to advanced disease. Sensitivity and specificity are comparable to current non-invasive (and mostly non-available) measures of NASH, but absence of PL have high NPV for NASH. Addition of the status of PL to common noninvasive scores of fibrosis improves their diagnostic yield. Our results, provided they are supported by larger studies, suggest that there is a role for addressing the status of portal lymphadenopathy in NAFLD patients and calls for examining the rule of ultrasonography for this purpose.

## Supporting information

S1 Dataset (XLSX)

## Abbreviations

NAFLD: Non-alcoholic fatty liver disease
NASH: non-alcoholic steatohepatitis
PL: portal lymphadenopathy
CT: computed tomography
MRI: magnetic resonance imaging
NAS: NAFLD activity score
F: Fisher’s
SC: spearman’s coefficient
HCC: hepatocellular carcinoma
IRB: institutional review board
SD: standard deviation
HB: hepatocytes ballooning
LI: lobular inflammation
PI: portal inflammation
ALT: alanine aminotransferase
TB: total bilirubin
INR: international normalized ratio
CRP: C-reactive protein
PLT: platelet
WBC: white blood cells
HgB: hemoglobin
IHD: ischemic heart disease
T2-DM: Type 2 Diabetes Mellitus
HTN: hypertension
OR: Odds ratio
PPV: positive predictive value
NPV: negative predictive values
APRI: ALT to PLT ratio
**FIB**-4: fibrosis-4 score
AST: aspartate transaminase
GGT: gamma-glutamyl transferase
ALK: alkaline phosphatase
